# Segmental testicular infarction: A case report

**DOI:** 10.1097/MD.0000000000047889

**Published:** 2026-02-28

**Authors:** Tong Yang, Wenxin Chen, Alimujiang Abdurexiti, Zhilu Gan, Ning Zhang

**Affiliations:** a Department of Urology, The Third People’s Hospital of Xinjiang Uygur Autonomous Region, Ürümqi, Xinjiang, China.

**Keywords:** scrotal pain, segmental, testicular infarction, testicular torsion

## Abstract

**Rationale:**

Segmental testicular infarction (STI) is a rare urologic disease with low incidence and rarely reported clinically; its clinical manifestations are complex and easily confused with other testicular diseases, leading to diagnostic difficulties. We reviewed a patient with unilateral STI and present the following report.

**Patient concerns:**

The patient presented to the hospital with sudden right scrotal pain for 14 hours.

**Diagnoses:**

Segmental testicular infarction.

**Interventions:**

The patient was diagnosed with STI. We gave the patient a subcutaneous injection of low-molecular-weight heparin and anti-inflammatory treatment.

**Outcomes:**

After 4 days of treatment, the testicular pain had subsided. When the patient was reevaluated 2 weeks later, his pain symptoms had subsided completely, and a scrotal color Doppler ultrasonography revealed that the right testicle had returned to normal.

**Lessons:**

Early diagnosis and timely treatment are crucial for improving prognosis. Color Doppler ultrasound is an effective tool for initial screening of testicular blood flow. For patients with testicular infarction, we should perform individualized treatment to preserve testicular function as much as possible.

## 1. Introduction

Segmental testicular infarction is a rare urologic disease with low incidence and rarely reported clinically, its clinical manifestations are complex and easily confused with other testicular diseases, leading to diagnostic difficulties. The disease usually manifests as acute testicular pain, which may be accompanied by scrotal swelling, tenderness and other symptoms, it even may affect the patient fertility.^[1]^ We reviewed a patient who with unilateral segmental testicular infarction, make the following report. The purpose of this study is to describe a young man case of testicular infarction, investigate its clinical signs, diagnostic techniques, and therapeutic approaches, and enhance medical professionals comprehension and management of this condition.

## 2. Case presentation

The 32-year-old man who complained of “sudden right scrotal pain for 14 hours” had never been hurt before. His right testicle was firm and sensitive when we palpated it.

**Figure 1.**
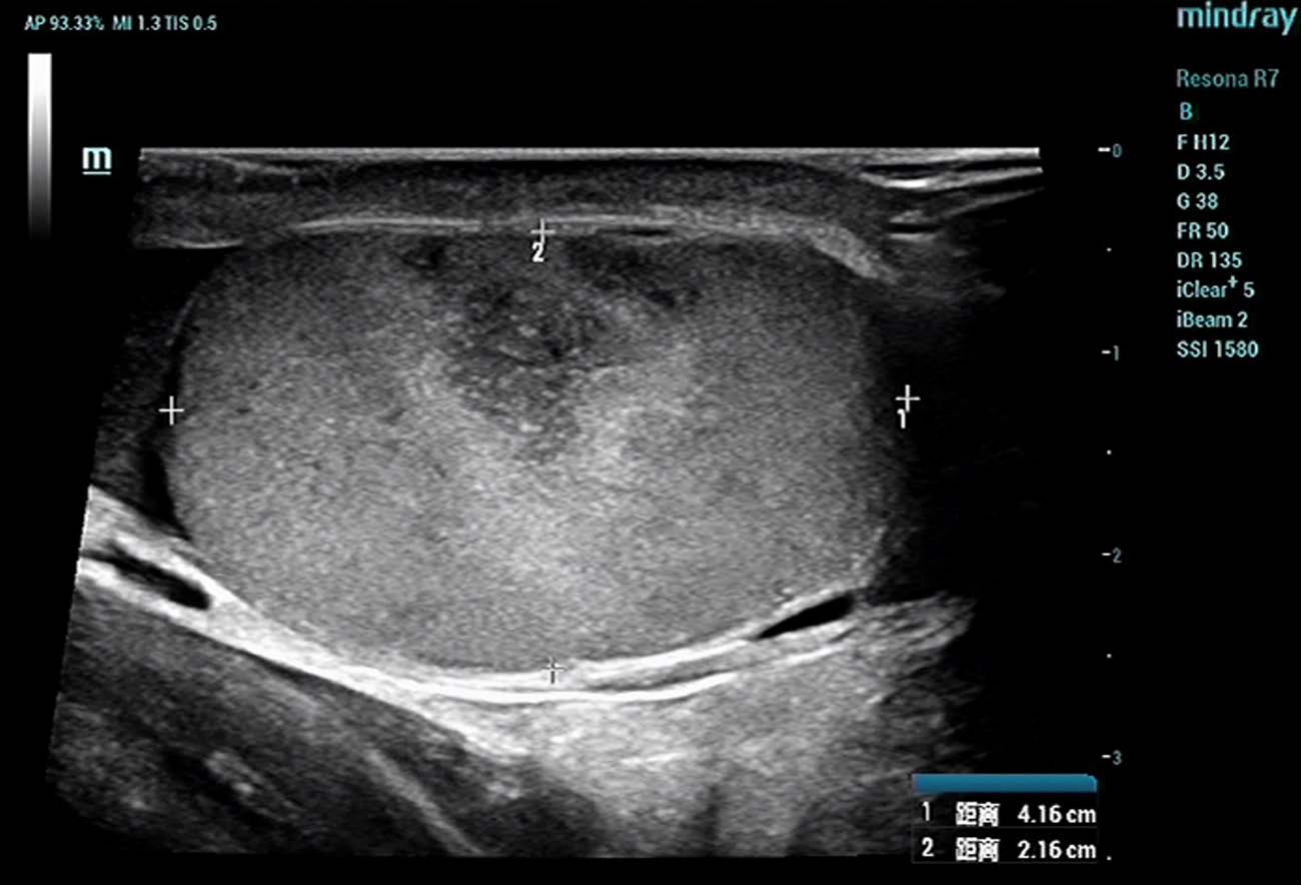
Revealed a homogeneous testis with an area of decreased parenchymal echogenicity.

**Figure 2.**
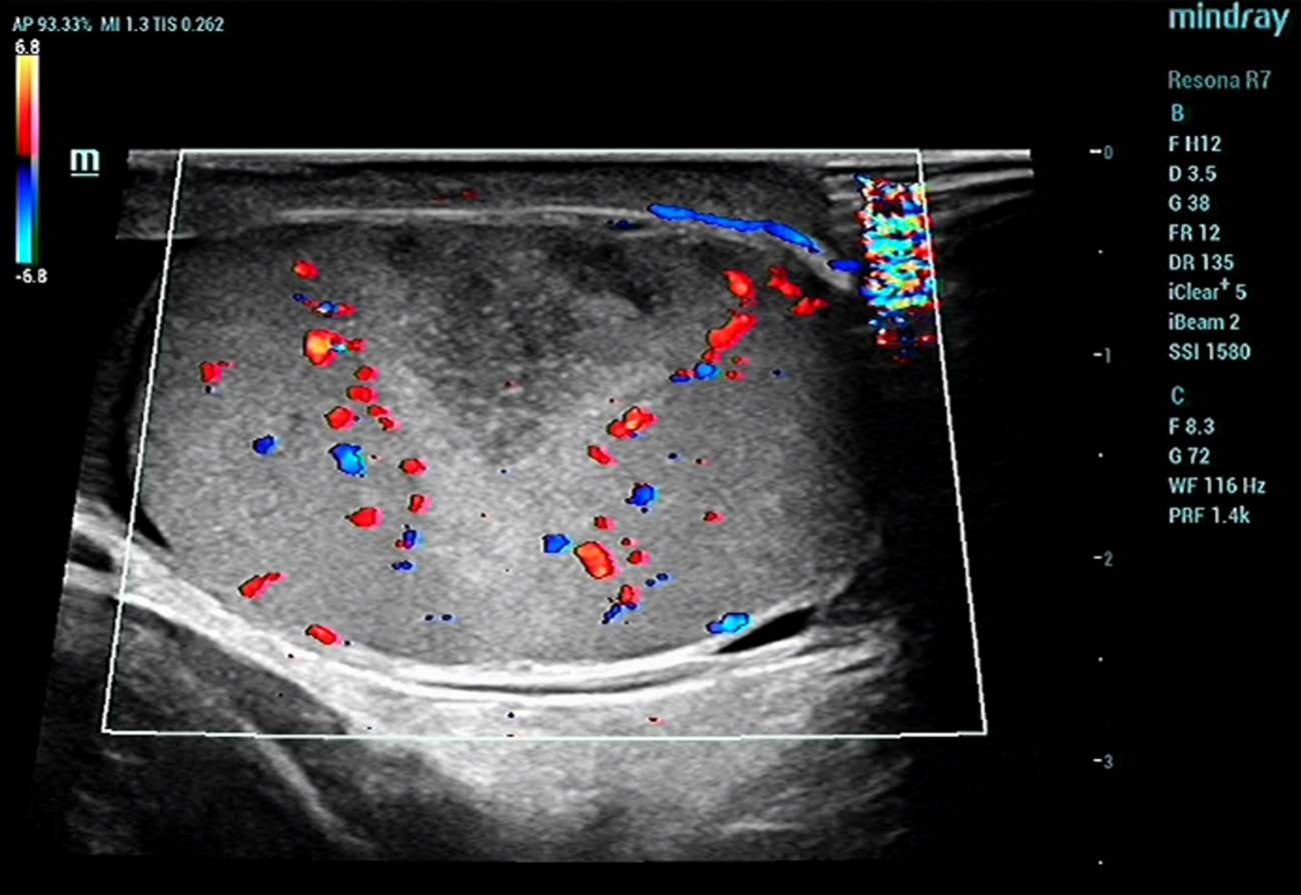
Color Doppler ultrasound of the testis that showed a hypoechoic area with an absent color flow in that area.

**Figure 3.**
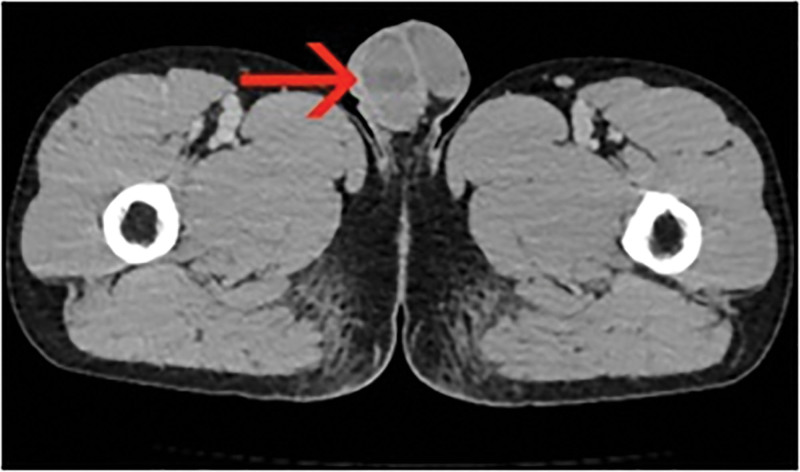
CT shows hypoechoic non-enhanced area in the right testis. CT = computed tomography.

**Figure 4.**
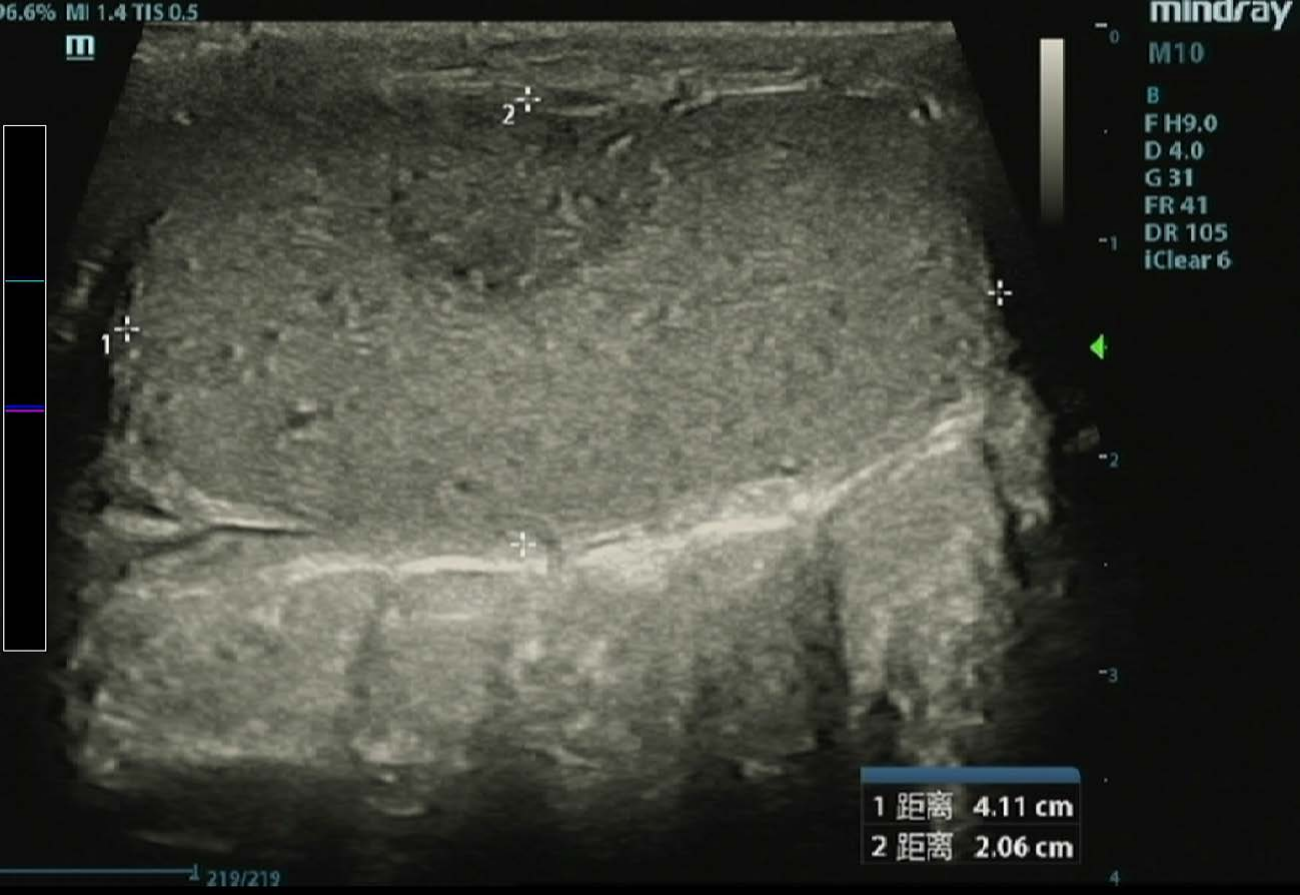
Ultrasound shows heterogeneous hypoechoic area with unclear boundary.

Blood routine examination indicated the following: leukocyte count of 9.55 × 10^9^/L (reference range: 3.5–9.5 × 10^9^/L), the neutrophil count is 8.07 × 10^9^/L (reference range: 1.8–6.3 × 10^9^/L), the level of interleukin-6 is 81.22 pg/mL (reference range: 0–7 pg/mL). Tumor markers and biochemical blood tests show normal results. Scrotal color Doppler ultrasonography shows a wedge hypoechoic segmental area in the right testis (Figs. 1 and 2), computed tomography shows a wedge hypodense area in the right testis that may indicate infarction (Fig. 3). After that, we gave the patient a subcutaneous injection of low-molecular-weight heparin, anti-inflammatory treatment and instructed him to stay in bed. Scrotal color Doppler ultrasonography revealed an inhomogeneous hypoechoic region with hazy edges on the right testicle after 4 days of treatment (Fig. 4), the testicular pain has subsided. When the patient was reevaluated 2 weeks later, his pain symptoms had subsided compeletly, and color Doppler ultrasound reveals an irregularly shaped area of infarction in the right testicle.

## 3. Discussion

The etiology of testicular infarction has not been fully elucidated, it may be associated with various factors. The most common cause is obstruction of the branches of the testicular artery, resulting in insufficient local blood supply to the testis and subsequent tissue necrosis. Several known causes include: First, vasculitis, which can lead to inflammatory stenosis or occlusion of the testicular arteries. Additionally, atherosclerosis and thrombosis can also cause testicular artery obstruction.^[2]^ Second, trauma or surgery in the groin area, which may damage the testicular artery or its branches, leading to impaired blood flow. Third, infectious diseases such as epididymitis and orchitis, which can induce local inflammatory reactions in the testicles, resulting in vasospasm or thrombosis. Fourth, anatomical abnormalities that may alter local blood flow dynamics in the testicles, increasing the risk of testicular infarction. Fifth, hematologic disorders such as sickle cell anemia and polycythemia vera, which increase blood viscosity and predispose to thrombosis, thereby obstructing blood flow.^[3,4]^ Some researchers have also identified a rare blood disorder, protein C deficiency, as being associated with the onset of testicular infarction.^[5]^

Initially, there is a reduction or interruption of blood flow in the testicular artery, leading to hypoxia of the testicular tissue. The seminiferous tubules exhibit edema, necrosis, and shedding of the spermatogenic epithelium, while the testicular interstitium demonstrates greater tolerance to ischemia and hypoxia. In the early stage, manifestations may include edema, vasodilation with stasis and congestion, and extravasation of blood cells. Clinically, patients often present with sudden, severe testicular pain and testicular enlargement. As ischemic time prolongs, testicular tissue damage worsens, blood flow within the testis ceases, and irreversible ischemia and infarction occur. Ultrasound examination may reveal wedge-shaped or round hypoechoic or heterogeneous intermediate echogenic lesions within the testis, most lesions show no blood supply, although the surrounding testicular parenchyma may exhibit increased blood flow and ring enhancement. In the late stage, testicular tissue undergoes fibrosis, the seminiferous tubules atrophy, interstitial inflammatory cells decrease, and testicular function is severely impaired.

Segmental testicular infarcts are rare and can be easily mistaken for other conditions, leading to frequent misdiagnosis. First, testicular torsion is more common in adolescents and children. It typically has a sudden onset with severe pain and is often triggered by identifiable causes such as trauma or vigorous exercise. On physical examination, the affected testis may be positioned transversely within the scrotum, exhibiting tenderness, increased tension, and noticeable thickening of the spermatic cord. Pain is often exacerbated when the testis is elevated. In contrast, testicular infarction is associated with relatively mild pain and may have an insidious onset. Some patients report no obvious precipitating factors. Ultrasound findings in testicular torsion include heterogeneous echogenicity of the testis and absence or significant reduction of blood flow signals. In testicular infarction, there is no blood flow signal in the localized infarcted area, while blood flow in the surrounding tissue may be normal or reduced.^[6]^ Second, epididymitis and orchitis are usually caused by bacterial infections and are characterized by swelling and pain in the testis and epididymis, often accompanied by fever and leukocytes in the urine. Ultrasonography typically reveals congestion or vasodilation of the testis with increased blood flow. Testicular infarction secondary to epididymitis is usually a chronic process associated with persistent pain. Third, testicular tumors may present as palpable masses within the testis. Ultrasound examination typically shows hypoechoic areas, but these usually exhibit blood flow signals, unlike the avascular hypoechoic areas seen in infarction. Measurement of alpha-fetoprotein and human chorionic gonadotropin levels can aid in confirming the diagnosis. Fourth, testicular abscess presents with testicular swelling and pain, often accompanied by fever and chills. Scrotal ultrasound reveals a hypoechoic area, which may show liquefaction. Fifth, varicocele can cause testicular pain, but this is usually chronic in nature.

When a patient presents with testicular pain, scrotal ultrasonography is an ideal imaging method. It can reveal a hypoechoic area in the testis with well-defined borders and a wedge-shaped morphology. Color Doppler ultrasonography typically shows an absence of blood flow signals in the infarcted region. Magnetic resonance imaging provides detailed information on the morphology and size of the infarcted area as well as changes in the surrounding tissues. On T1-weighted images, the infarcted area appears as a low signal, while on T2-weighted images, it shows a high signal. Additionally, there is usually no significant enhancement of the infarcted area after contrast administration. Computed tomography can also be used to diagnose segmental testicular infarction, but its sensitivity and specificity are relatively low. Some researchers have suggested that contrast-enhanced ultrasound may be more effective for diagnosing testicular infarction, as it provides better visualization of blood vessels, infarcted lesions typically demonstrate diffuse hypoenhancement or no enhancement.^[7,8]^

For patients with mild symptoms, conservative treatment can be used. It is generally recommended to observe the patient for at least 3 to 5 days and to determine whether further treatment is necessary based on the patient response and changes in symptoms. Antibiotics can prevent or control infection, while glucocorticoids and vitamin C help reduce the inflammatory response and oxidative stress, thereby protecting testicular tissue. Patients are advised to take bed rest, avoid strenuous activities, and closely monitor changes in the lesion.^[9]^ If the testis has been completely infarcted and has lost its function, or if there is severe infection or necrosis, orchiectomy may be required. Surgery can effectively remove necrotic tissue and prevent the spread of infection, however, maximum preservation of normal testicular tissue is essential. Long-term follow-up after hospital discharge is also crucial, the first follow-up is usually conducted 1 month after treatment to assess the testicular lesion and the degree of symptom relief. Subsequent follow-ups occur every 3 to 6 months and include ultrasound examinations to observe testicular blood flow and lesion changes, as well as tumor marker detection. Continuous follow-up should be maintained for at least 6 months to 1 year, until the lesion stabilizes or is absorbed and symptoms completely resolve.^[10]^

## 4. Conclusions

Early diagnosis and timely treatment are crucial for improving prognosis, color Doppler ultrasound is an effective tool for initial screening of testicular blood flow, while magnetic resonance imaging and contrast-enhanced ultrasound can provide more reliable evidence for definitive diagnosis. For patients with testicular infarction, we should perform individualized treatment, preserve testicular function as much as possible.

## Author contributions

**Data curation:** Tong Yang, Alimujiang Abdurexiti, Zhilu Gan.

**Writing – original draft:** Tong Yang.

**Writing – review & editing:** Tong Yang, Wenxin Chen, Ning Zhang.
